# Lipomeningocele with Tethered Cord Syndrome in an Adult: A Case Report

**DOI:** 10.31729/jnma.8220

**Published:** 2023-07-30

**Authors:** Soumya Pahari, Suraksha Thapa, Bibek Bhattarai, Bishal Pradhan, Paawan Bahadur Bhandari, Purushottam Baniya

**Affiliations:** 1 Department of Neurosurgery, Shree Birendra Hospital, Chhauni, Kathmandu, Nepal; 2 Nepalese Army Institute of Health Sciences, Sanobharyang, Kathmandu, Nepal; 3 People's Liberation Army Naval Medical University, Xiangyin Road, Shanghai, China

**Keywords:** *case reports*, *lipoma*, *spinal dysraphism*, *tethered cord syndrome*

## Abstract

Lipomeningocele is a closed neural tube defect characterized by the presence of spinal tissue within the spinal cord, with a junction between the spinal cord and a lipoma. It has a prevalence of 0.6 per 10,000 live births. A 29-year-old female presented to the outpatient clinic with complaints of weakness in both legs (more on the right) and tingling sensation and pain over her right thigh and legs for 1 year. She also mentioned swelling in her lower back since birth, which has been growing progressively since childhood. Magnetic resonance imaging of the spine revealed a low-lying spinal cord with the conus lying at the lower end of the L2 vertebral body. Spinal decompression with detethering of the cord and excision of the lipomeningocele was undertaken via a posterior midline incision. Prompt surgical intervention are crucial for symptom relief and prevention of neurological deterioration.

## INTRODUCTION

Lipomeningocele is a closed neural tube defect in which spinal tissue lies within the spinal cord having a junction between the spinal cord and a lipoma.^1^ Tethered cord syndrome refers to an abnormally low-lying conus medullaris tethered by a thickened filum terminale of different types of spinal dysraphisms including lipomeningocele.^2^ The prevalence of lipomeningocele has been found to be 0.6 per 10,000 live births.^3,4^ Around 50% of cases are asymptomatic at birth and symptoms progress as axial spine growth occurs. However, adult presentation is considered to be rare.^3,4^ Here, we present a case of a lipomeningocele with tethered cord syndrome who was asymptomatic since birth and presented at the age of 29 years with progressive neurological symptoms.

## CASE REPORT

**Figure 1:**
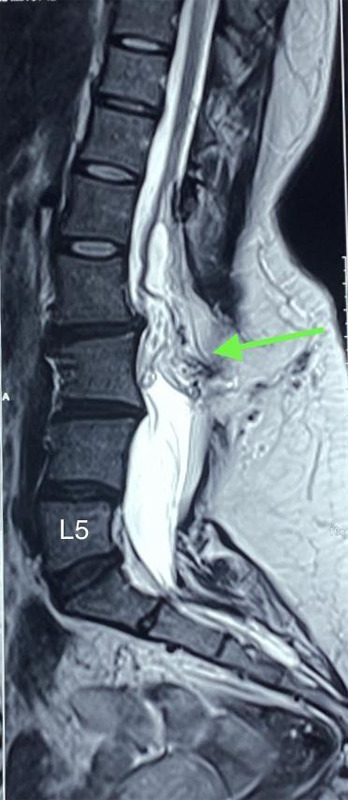
Contrast-enhanced MRI of the spine showing conus lying at the level of the lower end of L2 vertebra with herniation of epidural fat and small dural sac through defect in posterior elements at L3 level without herniation of neural components.

A 29-year-old female presented to the outpatient clinic with complaints of weakness in both legs (more on the right) and tingling sensation and pain over her right thigh and legs for 1 year. She also mentioned that she has had swelling on her lower back since birth, which has been growing progressively since childhood. There was no history of back pain, trauma, fever or significant weight loss. Her bowel and bladder habits were normal. Her past medical, surgical history and family history were non-contributory. On examination of the lower limbs, her power on the right lower limb was 3/5 and her power on the left lower limb was 4/5 according to the modified medical research council (MMRC) grade. Sensations and reflexes were normal in both lower limbs. Examination of the upper limbs and cranial nerves was normal. On local examination of the back, there was a midline swelling of size 7x5 cm in the lumbosacral area. It was soft in consistency with well-demarcated margins, immobile, non-tender and non-transilluminate. There were no skin pigmentary changes, tuft of hair, or skin dimpling. Her other systemic examinations and baseline laboratory investigations were normal. Magnetic resonance imaging (MRI) of the spine revealed a low-lying spinal cord with the conus lying at the lower end of the L2 vertebral body. Defects in the spinous process of L2 to visualized S2 vertebrae were present with nerve fibres with fat signal intensity (representing a neural placode) attached to the posterior dura at the L2 level. Herniation of epidural fat and small dural sac through the defect in posterior elements at the L3 level was present without herniation of neural components. The defect of the posterior element is covered by subcutaneous fat, with no communication of the dural sac with the external skin surface. There were no central spinal canal compromises or nerve root compressions (Figure 1).

A scanning MRI of the whole spine and brain revealed no other abnormalities. These features suggested a closed neural dysraphism with lipomeningocele and tethered cord syndrome.

Spinal decompression with detethering of the cord and excision of the lipomeingocele was undertaken via a posterior midline incision. Intraoperatively, a huge lipomatous mass extending from L2 to S1 spinal segments was observed. Spinal laminae from L2 to S1 were absent and the mass had an intradural extension. The posterior elements were fixed to form an abnormal bony mass that was placed laterally on the left side. The lipomatous mass was gradually dissected. The dura was opened and the intradural lipomatous mass was decompressed and the cord was untethered. The part of the mass adhered to the conus was left in situ to prevent injury to the neural tissue. A watertight closure of the dura was achieved.

The postoperative period was uneventful. On postoperative day 3, power on both lower limbs was 5/5. At 2 months follow-up, the patient was doing well and the symptoms of pain and tingling sensations had subsided.

## DISCUSSION

Tethering of the spinal cord has been associated with a diverse range of pathological conditions which includes a thickened and constricting filum terminal, intradural lipomas with or without an accompanying extradural component intradural fibrous adhesions, and the presence of myelomeningocele, among other factors.^3-5^ As opposed to meningomyelocele and meningocele which causes TCS in children, adult TCS like in our case is most commonly attributable to lipomeningocele, split cord malformation, and dermal sinus. The occult nature of the dysraphic state is one reason for not getting the desired clinical attention in these patients during their childhood.^6^

While lipomeningocele is typically diagnosed at birth and presents as a subcutaneous lipoma over the lower back, its presentation in adulthood is considered rare and if present is associated with TCS.^6,7^ In our case, the patient had been asymptomatic since birth and presented at the age of 29 years with progressive neurological symptoms, including weakness, tingling sensation, and pain in the lower limbs.

The surgical treatment for lipomeningocele with tethered cord syndrome involves the repair of lipomeningoceles, decompression of the spinal cord, and careful detethering.^8^ However, intradural lipomas as in this case tether the cord where they penetrate the dura. This requires circumcision of the dura and transection of the lipoma to separate the intra and extradural components.^9^ Managing the tethering effect of shortened nerve roots or arachnoid adhesions is challenging.

Complications like recurrent tethering, CSF leaks, infection, neurological deterioration, and scar tissue formation can arise, which may require further interventions or long-term management.^10^ The postoperative period, in this case however was uneventful, with the patient experiencing significant improvement in symptoms. By the third postoperative day, power in both lower limbs had fully recovered, and at the two-month follow-up, pain and tingling sensations had subsided.

Lipomeningocele with tethered cord syndrome in an adult patient case highlights the importance of considering lipomeningocele as a differential diagnosis in adults presenting with neurological deficits and the need for surgical intervention to alleviate symptoms and prevent further neurological deterioration. Additionally, the case emphasizes the significance of achieving a water-tight dural closure to minimize adhesion formation and the risk of retethering. This case report underscores the clinical presentation, surgical management, and positive outcome in an adult patient with lipomeningocele and tethered cord syndrome. While rare, lipomeningocele can present in adulthood, and prompt surgical intervention involving detethering and decompression are crucial for symptom relief and prevention of neurological deterioration.
